# Correlates of Persistent Smoking in Bars Subject to Smokefree Workplace Policy

**DOI:** 10.3390/ijerph6041341

**Published:** 2009-04-02

**Authors:** Roland S. Moore, Juliet P. Lee, Scott E. Martin, Michael Todd, Bong Chul Chu

**Affiliations:** 1 Prevention Research Center, Pacific Institute for Research and Evaluation, 1995 University Avenue, Suite 450, Berkeley, CA 94704, USA; E-Mails: jlee@prev.org (J.L.); smartin@prev.org (S.M.); mtodd@prev.org (M.T.); 2 Thomson Reuters, Healthcare, 5425 Hollister Avenue, Suite 140, Santa Barbara, CA 93111, USA; E-Mail: bong-chul.chu@thomson.com

**Keywords:** Smokefree workplace policy, tobacco control, bars, smoking behavior

## Abstract

This study’s goal was to characterize physical and social environments of stand-alone bars associated with indoor smoking despite California’s smokefree workplace law. In a random sample of 121 stand-alone bars in San Francisco, trained observers collected data on patrons, staff, neighborhood, indoor settings and smoking behaviors. Using bivariate (chi-square) and hierarchical linear modeling analyses, we identified four correlates of patrons’ indoor smoking: 1) bars serving predominantly Asian or Irish patrons, 2) ashtrays, 3) bartender smoking, and 4) female bartenders. Public health officials charged with enforcement of smokefree bar policies may need to attend to social practices within bars, and heighten perceptions of consistent enforcement of smokefree workplace laws.

## 1. Introduction

An extensive literature developed over the past 40 years has demonstrated the links between smoking and environmental tobacco smoke on the one hand and cancer, heart disease, and respiratory diseases on the other [1–4]. In order to reduce passive exposure to environmental tobacco smoke, public policies have increasingly restricted or banned the use of tobacco products in public places such as airplanes, schools, government facilities, and workplaces [5,6]. Workplace smoking bans in taverns are among the more controversial of these policies. Studies of workplace smoking bans have indicated that workplace smoking bans do effectively protect workers from the harmful effects of environmental tobacco smoke [7–9]. Bars, however, are often excluded from workplace smoking bans, due perhaps to deeply-entrenched social norms connecting smoking with drinking, the main business of bars [10]. Yet bar workers are among those most affected by tobacco exposure in workplaces not subject to smokefree laws [11–15].

California Assembly Bill 13 (AB 13), the Smokefree Indoor Workplace Act, was passed in 1994 and applied to bars statewide in 1998 [16–18]. Framed as worker protection legislation, the smokefree workplace act, now officially California Labor Code Sections 6404.5, 2698–2699, prohibits smoking in enclosed spaces in places of employment, including bars and restaurants. While businesses that are solely operated by the owner are eligible for exemption, in practice few bars meet this requirement, as janitors, musicians, or even “stock-holding” bartenders are considered employees [19]. Enforcement is the responsibility of local agencies, usually police or sheriffs, in coordination with county health departments. Progressive fines of $100, $200, and $500 may be followed by referral to the California Occupational Safety and Health Administration, which has imposed higher fines for continuing noncompliance [16,19].

Overall, the implementation of this law has been successful; 95.4% of respondents on the 2002 California Tobacco Survey reported that there was no smoking in their workplace, compared to 35% in 1990 [20]. Compliance with the smokefree workplace law in bars, however, has not been as complete. Stand-alone bars—those bars not connected to a restaurant or hotel—are less compliant than other bars. For example, rigorous compliance checks in Los Angeles County found patron compliance rates in stand-alone bars to be 45.7% in 1998, increasing to 75.8% in 2002 [21]. Both theoretically and practically, it is important to establish the factors responsible for successful or incomplete prevention policy implementation. The present study sought to identify characteristics of the social and physical environments of bars that were associated with compliance or noncompliance with the smokefree workplace law.

## 2. Methods

The two-year project (2002–2003) used highly-structured naturalistic observations together with semi-structured interviews to collect both quantitative and qualitative data. The researchers compiled a census of stand-alone bars throughout San Francisco based on California Alcohol Beverage Control data and local entertainment guides. The list was verified through a scouting survey (described below); establishments were excluded that were judged not to be stand-alone bars (e.g., nightclubs where the primary activity was live music, or large pool halls where drinking was of secondary importance). From the final census of 345 stand-alone bars, a random sample of 120 bars was generated. Whereas four of the sample bars were replaced due to closures during the data collection period, one reopened and was re-included. The final sample of 121 bars reflected the range of such establishments throughout the city, including all types identified in the literature on bars such as service bars, neighborhood bars, and pick-up bars [22].

### 2.1. Scouting

Field staff conducted an on-site survey of each potential location to establish whether it still existed; to confirm the addresses, to establish whether or not the bars met the sample criteria, to document conditions related to safety, patron demographics, and hours for future observations, and to document general conditions, including bar characteristics and indications of smoking inside the bar. This survey was conducted using a survey instrument that we designed, programmed and then loaded onto handheld computers (personal digital assistants, or PDAs) [23].

### 2.2. Observations

Pairs of trained observers conducted four sets of unobtrusive observations lasting approximately one hour in each of the 121 sample bars. Each bar was visited at least once during each of three time periods, designated as happy hour (5–8 pm), evening (8–11 pm) and late night to closing (11 pm-2 am). Additionally, bars were visited at least three different days of the week, including one weekend night (Friday or Saturday). Field staff members were instructed to look for both clear and circumstantial evidence that persons within the bar were not complying with the smokefree ordinance. Data from a total of 479 observations were analyzed below; safety concerns and frequent closures resulted in five establishments represented by only three instead of four observations.

### 2.3. Measures

The research team designed an observational data collection instrument using Pendragon form creation software [24]. Following extensive pretesting, the form was loaded onto the observers’ PDAs. Field staff then electronically transmitted the data from these forms to a central database.

Using the PDA form, field staff collected data on distinguishing characteristics of the bars and their patrons, including age, ethnicity, gender, apparent socioeconomic status, and number of patrons. More specifically, certain items were asked as yes/no questions, such as the presence of a patio, ashtrays, observed illicit drug use by patrons (e.g., smoking marijuana), apparent underage patrons, and if the weather was pleasant for smoking outside. Occurrences of other characteristics were counted and recorded in the instrument and subsequently recoded into binary variables (present or absent) for analytical purposes. These recoded items include the number of visible tobacco advertisements, tobacco company-provided bar supplies, glamorized images of smoking (such as posters of movie stars holding lit cigarettes), and no smoking signs. The number of bartenders and rooms were recoded into binary variables representing one versus more than one of their respective items. Observers were asked to determine what type of drink (beer, wine, liquor) patrons were predominantly drinking. Because beer was most frequently recorded as the predominant drink at the time of observation, this variable was recorded and analyzed as predominantly beer (yes or no). How busy the bar seemed was rated on a scale of one to five (representing the ratio of the number of patrons to the capacity of the bar). Bartender gender was assessed by the observers both in the observation instrument and in their narratives. The day of week and the time of the observation were also recorded by the observers. In our analyses, we used two sets of dummy coded variables, one coding for all seven days of the week and one set representing either weekend (Friday and Saturday) nights or weeknights (Sunday through Thursday).

Observers were instructed to identify patron and staff ethnicity or membership in definable groups based on appearance as well as overheard languages spoken and/or accented English. For example, Irish patrons were identified by their accents and ethnic markers in the décor and setting of the bar (such as music, signage, posters and photographs). While patronage in the majority of bars was mixed or heterogeneous, many other bars were observed to serve primarily one type of patron. The four primary identifiable types of patrons observed in the San Francisco sample bars were categorized either by their ethnicity (Asian, Irish labor migrants, Latinos) or their sexual preference (LGBT: lesbian, gay, bisexual and transgender patrons). The assessments of the observers’ classifications were then double-checked by consulting guides to bars in Northern California weekly entertainment newspapers and newspapers targeting specific ethnic groups (such as the *Irish Herald* and *Asian Week*), as well as Internet bar guides specifically describing the clientele in many of the bars that were included in our sample. It should be noted that the majority of bars in the sample catered to multiple types of patrons in which no single type of patron predominated; such bars were assigned the patron category of “other”.

The primary variables of interest, indoor smoking by bar patrons and by bar staff, were defined as any observed cigarette smoking whatsoever. We operationalized noncompliance with the smokefree workplace ordinance as positive any time either observer witnessed smoking of a cigarette inside the establishment. Lighting up on the way out of the bar was counted as indoor smoking. However, if a room contained less than four walls and was open to the outside, and/or was roofless (e.g., a patio), smoking was noted but counted neither as indoor smoking as defined by the smokefree workplace law nor as smoking lawfully outside the bar [19]. Smoking outside, either on the street, sidewalk, or parking lot adjacent to the bar, or in a patio open to the outside was not counted as indoor smoking.

In addition to filling out the checklists, the observers produced brief but detailed fieldnotes for each observation describing the physical environment, bar staff and patrons, as well as reporting on staff/patron interactions and in particular observed behavior related to compliance or noncompliance with the smokefree law [25]. In addition to serving as invaluable sources of qualitative data on the social dynamics within bars [26] and rationales for indoor smoking by bar staff and patrons [27], these data could be used to assess discrepancies in the survey data. The observers worked in pairs and were permitted to confer on their findings, but each submitted a separate record for both PDA and narrative data on each observation.

Additional measures of neighborhood characteristics including median household income, proportion of the population below the poverty line, and percentage of foreign-born residents in the neighborhood were also collected. These census block-level data were drawn from 2000 U.S. Census databases and linked to the geocoded locations of the randomly selected bars.

### 2.4. Analysis

In our primary analyses, the variables recorded by the observers were treated as being either at the observation (or occasion) level or at the bar level. The first step of data reduction for the structured checklist condensed both observers’ data conducted during a single observation occasion into one occasion-level set of variables, using the decision rule that if either of the observers saw a behavior, it was coded as present. The inter-rater reliability on the checklist items ranged from 0.60 on poorly defined variables (such as how busy a bar seemed) to 0.99 on such items as the presence or absence of a security guard (bouncer) and physical features of the bar; the mean agreement was 0.86. When observers disagreed about whether they saw smoking, the source of the discrepancy was usually the fact that they were asked to circulate through the bar at different times, thus spotting different transient phenomena. For variables that remained consistent across observations, such as predominant patron ethnicity and sexual preference, all observations conducted at each bar were compared and bar-level variables were constructed. Descriptive statistics (including Pearson’s χ^2^ for discrete variables and *t*-tests for continuous variables) were then used to compare the compliant and noncompliant bars on all observed variables recorded on the checklists and to assist in building the multilevel regression models described below.

The data analyzed here have an inherently multilevel structure with repeated observations (Level 1) nested within bars (Level 2). Also, it is reasonable to expect observations from the same bar to be more similar to each other than they are to observations from other bars. The multilevel structure and potential for non-independence of observations from the same bar suggest that multilevel regression (also known as hierarchical linear modeling) techniques are best suited to these data. Failure to account for potential dependence among observations within bars can lead to biased tests of regression coefficients and thus erroneous conclusions regarding their significance. Accordingly, we used multilevel logistic regression models (estimated using SAS PROC GLIMMIX) for primary analyses.

Our initial multilevel regression model was a null model (identified as Model 1 below) that included only a random Level 1 (bar-specific) intercept, which allowed us to estimate the variance in the outcome variable, patron smoking (i.e., policy non-compliance). We then estimated a second model (Model 2), which comprised the intercept and bar-level covariates only. The final full multilevel model (Model 3) included the intercept and bar-level covariates from Model 2 along with occasion-level variables as covariates of patron smoking. To obtain estimates of purely within-bar relations between the occasion-level covariates and the outcome, we group-mean centered the occasion-level predictors. This approach minimized the influence of between-bar variation in the occasion-level predictors on the estimates of within-bar associations [28]. As we found no significant random components for the Level 1 slopes (i.e., relations between occasion-level predictors and the outcome did not vary across bars), the final model had only a random intercept component (i.e., only the Level 1 intercepts or log odds of the bars’ mean levels of compliance were allowed to vary across bars). This final model allowed for simultaneous examination of how bar characteristics and situational factors were related to the likelihood of observed indoor smoking.

## 3. Results

### 3.1. Univariate and Bivariate Analyses

Preliminary univariate analyses of the survey data indicated that indoor bar patron smoking occurred in 30% of the observations (see Table 1). Overall, smoking was directly observed in 49.6% of sample bars during at least one of the four observation visits. As noted in Table 1, 9% of bars had predominantly Asian patrons, 5% had predominantly Irish patrons, 7% had predominantly Latino patrons, 10% had predominantly LGBT, and in the remaining 69% of bars no ethnicity or sexual orientation predominated. On most observation occasions, only male bartenders were on duty (54%), on about a third (35%) of the occasions only female bartenders were on duty, and on 11% of the occasions mixed-gender bartending staff were working. Ashtrays were visible inside the bars on 24% of the observation occasions.

To determine which covariates would be included in our primary analyses, we conducted preliminary bivariate chi-squared tests examining associations between bar- and occasion-level measures of the bar environment and patron smoking. Variables found to be significantly related to patron smoking at this stage were retained for use in the multilevel regression models.

Among the elements of the bar environment and neighborhood demographics that were assessed, only bar type (i.e., predominant ethnicity and sexual orientation) was significantly associated with patron smoking in the sample bars (see Tables 2 and 3). Elements such as having tobacco products for sale or tobacco company logo items (for example, coasters and bar napkins), having posted “no smoking” signs or “glamorized” images of smoking (for example, posters of movie stars smoking cigarettes), visibility from the street (measured by location on main thoroughfares) or from the sidewalk (measured by location in a dense business area), and neighborhood income level, however, were not related to patron smoking.

Among the features expected to vary across observation occasions at the same bar, bartender gender, employee smoking, and the presence of ashtrays were all related to patron smoking (see Table 4). Patron smoking was most likely to be observed on occasions when only female bartenders were present, occasions when employees were also smoking, and occasions when ashtrays were visible in the bar. Variables such as day of the week and time of day, as well as indications of staff laxity towards legalities including observed illicit drug use or the presence of apparent underage patrons in the bars, were not related to patron smoking.

### 3.2. Multilevel Logistic Regression Analyses

The null model (Model 1) showed that the mean probability of indoor smoking differed significantly across bars (see Table 5). In line with the findings from the bivariate analyses, the next model estimated (Model 2) included only the intercept and bar type as predictors of patron smoking. Bar type was represented in the multilevel regression model by three dummy variables coding for predominant patron ethnicity (Asian vs. non-Asian, Irish vs. non-Irish, Latino vs. non-Latino) and one dummy variable coding for predominant patron sexual orientation (LGBT vs. non-LGBT). Results from this analysis showed that bars serving predominantly Asian and Irish patrons were more likely to have patrons smoking inside the bars (i.e., more likely to be out of compliance with the smokefree workplace law; see Table 5). The substantive findings regarding associations between the bar type variables and mean likelihood of patron smoking did not change after adding occasion-level covariates identified in the bivariate analyses (Model 3). The associations between occasion-level variables and indoor smoking seen in the bivariate analyses also emerged in the multilevel regression analyses. On occasions when ashtrays were present in bars and occasions when bar employees were smoking, patrons were more likely to be smoking than on occasions when ashtrays were not present and on occasions when employees were not smoking. Also paralleling the bivariate findings, patron smoking inside bars was relatively more likely on occasions when only female bartenders were on duty (see Table 5). Using techniques described by Snijders and Bosker [29, pp. 225–227], we calculated measures of the percent variance in the outcome accounted for by the model predictors. The bar-level only model accounted for 17.4% of the variance in the patron smoking variable, while the full multilevel model accounted for approximately 27.1% of the variance in this variable.

### 3.3. Supplemental Analyses

Bivariate analyses of the data from our sample of 121 randomly selected stand-alone bars showed a significantly higher prevalence of smoking inside bars with predominantly Asian and Irish patrons and relatively lower prevalence of indoor smoking in bars frequented by predominantly Latino or LGBT patrons. To determine if these findings could be replicated, we subsequently conducted an additional round of observations at 72 bars serving Asian, Irish, Latino, and LGBT patrons that were part of the original universe of San Francisco bars but not part of the randomly chosen group of 121 bars. Each of the 72 bars was visited once by a pair of observers. A similar pattern of associations between bar characteristics and smoking was observed in this additional set of bars. Specifically, indoor smoking was observed in 10 of 17 bars serving Asian patrons and 10 of 18 bars serving Irish patrons. Conversely, indoor smoking was only observed in nine out of 28 locations serving LGBT patrons and in none of the nine bars serving Latinos.

## 4. Discussion

This study found that while 70% of observations in stand-alone bars in San Francisco revealed no evidence of indoor smoking, in a subset of bars indoor smoking continued to be observed, at least some of the time. The findings presented here indicate that, rather than external factors such as time of day, day of the week, or location, noncompliance with the law appears to be related to internal features or aspects of bar culture. The actual incidence of smoking across all the observation periods was approximately 30%. Any infraction whatsoever (such as lighting up on the way out of the bar) was counted as indoor smoking. The percentage of bars within which the majority of patrons were observed smoking (what we term “endemic smoking”) was much smaller. Our findings could be compared with those of Weber *et al.* [21] for Los Angeles County (from 45.7% compliance in freestanding bars in 1998 to 75.8% in 2002) and of RTI International’s [30] report on bars in New York following its Clean Indoor Air Act policy enactments (as low as 14.8% noncompliance visible in bars, although the odor of smoke, some of which may have wafted in from outside, was perceptible in 25% of bars 12 months following enactment). In comparison with those studies, it seems that San Francisco has a higher rate of noncompliance with the smokefree workplace law. Our observers, however, spent an hour per visit, on four separate occasions, which is much longer than most county health inspectors or the researchers in the aforementioned projects were able to spend in their compliance checks, thereby making direct comparison with their data problematic.

The correlation between ashtrays and smoking indicates the importance of behavior in signaling the bar’s regard for the law. Provision of ashtrays to patrons tacitly signals approval of smoking; staff smoking may enhance this signal, and further model behavior for patrons. The findings relating to the gender of bartenders indicates that social control may be an issue; female bartenders may be less able or less willing to enforce the nonsmoking laws. We hypothesize that it is related to the gender-based power differentials in bars – women (especially low-SES immigrant women, of whom there were many in our study) may find it more difficult than men to refuse the requests of their male patrons, or even more so of their predominantly male employers.

Patron characteristics were strongly related to the likelihood of smoking in the bars. While smoking was significantly less likely to be observed inside bars serving predominantly LGBT and Latino patrons, bars serving predominantly Asian and Irish ethnicity patrons were significantly more likely to be smoky. Bars serving a heterogeneous mix of patrons were not found to have significant higher rates of smoking than other, more identity-specific, bars. These findings suggest the importance of social cohesion and intragroup dynamics in terms of the likelihood of compliance with the smokefree law [26].

The findings on ethnicity and compliance are compelling. California is home to over 11 million Latinos or Hispanics and four million Asians or Asian-Americans (respectively 32.4% and 12.3% of the state population) [31]. Statistics from World Health Organization surveys in the late 1990s [32] show smoking in Asian countries of origin to be much higher than in California. This suggests that Asian bar smoking may be supported by recent immigrants from China and Korea (where over 60% of men smoke), and Vietnam (over 70% male smoking prevalence). Smoking rates in Latin American countries of origin vary but tend to be lower than in Asia (51% of men between the ages of 18 and 65 in Mexico smoked). In the last two decades, a major influx of people from Ireland has entered U.S. urban centers, including Los Angeles and San Francisco [33]. Ireland features lower prevalence among adult males (32%) – but this rate is still higher than in California. There is very little literature specifically on bar smoking behavior in any of the regions mentioned above. While Asians and Latinos smoke less than the overall U.S. population, and smoking may not correlate with drinking in general, there may be an association between smoking and heavy drinking for Asian and Irish bar-goers, although not for Latinos.

The findings of high compliance with the smokefree law among gay bars were surprising given the high rates of smoking among this group [34,35]. Reasons for high compliance with the law in gay bars raised by gay bartenders and patrons in our interviews include not attracting police attention by adhering to the law, and early highly visible enforcement efforts [36].

The correlation between non-smoking in bars and some type of homogeneity of patrons, whether by ethnicity or sexual orientation, suggests that group dynamics play an important role in smokefree bar policy compliance. The finding of a significant relationship between staff smoking and any smoking in bars indicates the impact of this influence on compliance with the smokefree workplace law. As we have reported elsewhere, patrons and staff both pointed to the bartender as a focal point of the bar, moderating the flow of conversation and social interactions as well as the flow of alcohol within the bar environment [26]. Female bartenders may either be less able to control their patrons smoking, or may feel more pressure to smoke with patrons as an expected part of socializing [37].

Patrons and staff alike described family-like relationships among regulars and between regulars and staff, including dates, dinner parties, barbecues, and invitations to weddings. In the bar the line between staff often blurs as regulars “help out” the bartender behind the bar, often eventually becoming paid staff themselves [26]. Such tight-knit networks of bar regulars may serve to create powerful social systems which could profoundly help or hinder staff efforts to comply with the law [38–40]. Tobacco research has indicated that heavy smoking subcultures may develop within social environments of lighter or no smoking [41]. Findings from this San Francisco study seem to bear this out; although within four years most bars in the city had converted to full or partial compliance, in a significant subset of bars smoking appeared to continue to be highly normative, possibly representing the retrenchment of a smokers’ culture in some bars. The relationships between bartenders and regular and visiting patrons appear to have reinforcing effects in compliance or noncompliance. Other social psychological concepts relevant to compliance include self-selection into groups and reinforcement for smoking by means of cue reactivity, processes that are not limited to adolescent smokers [42,43].

The association between alcohol and tobacco use is longstanding, even as the specific physiological and biochemical processes linking them are not fully understood [44–46]. It is well-established that people have been smoking in bars for decades. There are profound symbolic relationships, as well as chemical connections, between smoking and leisure, smoking and relaxation, smoking and sex, and well-entrenched norms about bars and leisure, bars and relaxation, and bars and sex [47]. Thus, it should not be surprising that bar-goers are reluctant to change the habit of smoking in bars.

### Limitations

This study took place in California, where the smokefree workplace law extension to bars has been in place over ten years and where, additionally, reduced smoking and tobacco control policies have been becoming normative, compared to other parts of the country. San Francisco is, moreover, known to be socially progressive and yet at the same time to celebrate individuality and freedom of expression. This cultural framework may shape the way individuals and social groups respond to the smokefree bar ordinance. A similar study based in another state, or even another California city, may show different results.

Another potential limitation concerns the reliability of the observational data. The characteristics of the people in these bars such as age, ethnicity, or sexual orientation are problematic to determine through observation only. This could introduce bias to our results because they are not self-reported or documented through reliable records. The use of accent or face features does not always convey ethnicity. Reliability may have been affected by the observers’ conferring about some variables. However, most often these discussions concerned fixed variables which, due to poor visual conditions, may have been difficult for one observer to see, such as no-smoking signs and images of smokers. Moreover, because some indoor smoking behavior was transient, and the observers were separated at times, we expected that there would be instances where one of them saw someone smoking and the other, in the restroom or in another part of the bar, might miss seeing this. Due to such working conditions, the researchers met regularly with the field observers to discuss such issues and to clarify decision rules and coding processes. Therefore, if either of the observers reported observing indoor smoking, we felt confident about their observations.

The absence of significant relationships between many of the study variables and observed noncompliance with the smokefree workplace act should be interpreted with caution. For example, in a dense urban setting it is difficult to assess the socio-economic status (SES) of an area from census data, where average incomes vary greatly from block to block and other indicators such as the SES of patrons may be difficult to assess using observational data.

Another issue that was difficult to assess from the observational data was the impact of enforcement of the law. Enforcement of California’s smokefree workplace law is determined and implemented at the city and county level of government. In San Francisco, enforcement has been mostly complaint-driven and most effectively followed up through a combination of inspections and legal proceedings; but unless an enforcement incident took place during the time of an observation, the data presented here would not show the effect of enforcement activities. The impacts of socioeconomic status and of enforcement on bar smoking are, therefore, better addressed through qualitative methods, and as such will be discussed in separate publications. In a follow-up study, the findings reported in this paper were reinforced in interviews with public health and law enforcement officials in the region. They repeatedly stated that they are well aware of differential patterns of compliance with the smokefree workplace law in bars throughout the city. For example, they specifically targeted bars serving Irish patrons with a mailed reminder (picturing the facades of many of these Irish bars) that indoor smoking was no longer permitted; they then issued expensive citations to one of the most egregious violators [48].

### Implications for policy makers and enforcers

California is recognized as a leader in tobacco control policy, and its smokefree workplace law compliance in most kinds of establishments (including restaurants and restaurant-bar combinations) is exemplary. However, within the category of stand-alone bars, compliance is still incomplete. As smokefree bar ordinances are increasingly passed throughout the country, other states look to California for models of how to support these laws after they are enacted. Implications for other states and municipalities considering workplace smokefree acts are that normative shifts and corresponding compliance with smokefree workplace policies are likely to be successful in offices and restaurants; however, additional efforts, including policy reinforcement through enforcement, may be needed to solidify the normative shift to smoking outside of bars, and stand-alone bars in particular.

Our primary recommendation to policy enforcers is to instill in bar owners the idea that if indoor smoking is not allowed in their bars as well as those of their competitors, a level playing field will ensure that they will not lose customers. This has indeed been shown by the majority of economic studies of successful smokefree workplace policies in bars and restaurants throughout the country and the rest of the world. Compliance is far more likely when the owners, staff, and patrons perceive that that the policy will be enforced, which may require well-publicized enforcement efforts by the relevant agencies [48]. Targeting factors identified here, including visible ashtrays, categories of bar patrons highly likely to be smoking in bars, and bartender smoking, would help make this enforcement process more efficient.

The findings of this study associating smoking inside stand-alone bars with: (1) specific patron types, (2) the presence of ashtrays, (3) bartender smoking, and (4) female bartenders serving male patrons, may together suggest that the public health officials and other personnel who are charged with enforcement of smokefree bar policies must devote attention to the internal policy-setting practices and social interactions of bar owners, managers, and bartenders. The successful exemplars of smokefree workplace legislation in California and other states, counties, and communities in the U.S. and abroad have clearly not only reflected but also affected shifts in cultural beliefs and practices related to smoking. Despite imperfect compliance with California’s smokefree workplace law, the observations conducted in this study indicate that overall, bar staff as well as patrons are exposed to dramatically less secondhand smoke than prior to the extension of the policy to bars in 1998.

## Figures and Tables

**Table 1.** Descriptive information on the sample (N = 121 bars, n = 479 observations).

**Variables**	**Proportion**	**SD**
**Dependent Variable**		
Patron smoking observed	0.30	0.46
**Covariates Bar-level measure**		
Asian patrons	0.09	0.28
Irish patrons	0.05	0.21
Latino patrons	0.07	0.26
LGBT patrons	0.10	0.30
**Observation-level measure**		
Ashtray present	0.24	0.42
Male bartenders only	0.54	0.50
Female bartenders only	0.35	0.48
Female and male bartenders	0.11	0.30
Employee smoking observed	0.14	0.35

**Table 2.** Chi-square tests for associations between bar environment variables and patron smoking (noncompliance with AB 13).

	***n***	**% non-compliant**	***X^2^***	**p-value**
**Tobacco product ads**			0.01	0.94
Yes	54	50.0		
No	67	49.3		
**Tobacco company logo bar supplies**			0.03	0.86
Yes	27	48.1		
No	94	50.0		
**Glamorized images of smoking**			2.00	0.16
Yes	24	62.5		
No	97	46.4		
**No smoking signs**			3.62	0.06
Yes	113	51.3		
No	7	14.3		
**One-room bar**			0.11	0.74
Yes	83	50.6		
No	38	47.4		
**Has patio**			0.00	0.98
Yes	12	50.0		
No	109	49.5		
**Predominant patron type of bar**			16.57	<0.01
Asian	11	100.0		
Irish	6	83.3		
Latino	9	33.3		
LGBT	13	38.5		
Other	82	43.9		

**Table 3.** Means, (standard deviations), and *t*-tests for comparisons of U.S. Census block characteristics for compliant and non-compliant bars.

**Census block measure**	**Compliant bars**	**Non-compliant bars**	***t***	***p*-value**
**Median household income of census block (in $)**	45198 (17553)	51338 (20697)	−1.76	0.08
**% Residents living below poverty**	14.9 (8.9)	14.5 (13.5)	0.18	0.86
**% Residents who are foreign born**	41.6 (14.0)	35.7 (19.1)	1.93	0.06

**Table 4.** Chi-square tests for bivariate associations between patron smoking (noncompliance with AB 13) and night-level variables, ignoring within-bar clustering.

**Variables**	***n***	**% non-compliant**	***X*^*2*^**	**p-value**
**Bartender gender**			15.51	<0.01
Only men	252	22.6		
Only women	165	40.6		
Both	49	28.6		
**Ashtrays present**			235.58	<0.01
Yes	113	87.6		
No	366	12.0		
**Employee smoking**			107.13	<0.01
Yes	69	82.6		
No	410	21.0		
**Day of week**			1.42	0.96
Sunday	50	28.0		
Monday	70	30.0		
Tuesday	51	31.4		
Wednesday	90	33.3		
Thursday	75	32.0		
Friday	96	36.5		
Saturday	47	31.9		
**Weekend**			0.63	0.43
Yes	143	35.0		
No	336	31.3		
**Time of night**			1.97	0.37
Happy Hour (5–8pm)	135	28.1		
Evening (8–11pm)	217	32.7		
Late Night (11-2am)	127	36.2		
**Conditions outside pleasant**			2.39	0.12
Yes	453	31.6		
No	26	46.2		
**Beer was main drink**			0.39	0.53
Yes	225	33.8		
No	254	31.1		
**More than one bartender**			0.94	0.33
Yes	297	34.0		
No	172	29.7		
**Illicit drug use**			0.23	0.63
Yes	15	26.7		
No	464	32.5		
**Apparent underage patrons**			2.10	0.15
Yes	24	45.8		
No	455	31.6		

**Table 5.** Parameter estimates and standard errors (SE) for multilevel regression models predicting patron smoking.

**Fixed effects**	**Model 1 Random intercept-only (null) model**		**Model 2 Bar-level predictors model**		**Model 3 Full multilevel model**	
	**Coefficient**	**SE**	**Coefficient**	**SE**	**Coefficient**	**SE**
Intercept	−1.81†	0.29	−2.08	0.35	−2.58†	0.45
**Bar-level predictors**						
Asian patrons			3.15†	0.94	4.38†	1.24
Irish patrons			4.15†	1.35	5.59†	1.74
Latino patrons			0.09	1.13	0.54	1.44
LGBT patrons			−0.56	0.98	−0.33	1.24
**Observation-level predictors^a^**						
Female bartenders					1.38†	0.40
Male and female bartenders					1.13*	0.55
Ashtray					4.64†	0.52
Employee smoking					1.69†	0.47
**Variance components**						
τ_00_	7.98†	1.38	7.24†	1.29	12.73†	2.19
Overdispersion parameter	0.40	0.03	0.40	0.03	0.29	0.02

^a^ predictors are group-mean centered

* significant at p < 0.05

† significant at p < 0.01

## References

[1] Barnoya J, Glantz SA (2004). Secondhand smoke: The evidence of danger keeps growing. Am. J. Med.

[2] National Cancer Institute (1993). Respiratory Health Effects of Passive Smoking: Lung Cancer and Other Disorders: The Report of the.

[3] (1999). Health Effects of Exposure to Environmental Tobacco Smoke: The Report of the California Environmental Protection Agency.

[4] U.S. Department of Health and Human Services (1989). Reducing the Health Consequences of Smoking: 25 Years of Progress. A Report of the Surgeon General (DHHS Publication No. (CDC) 89–8411.

[5] Osinubi OY, Slade J (2002). Tobacco in the workplace. Occup. Med.

[6] Sorensen G, Glasgow RE, Topor M, Corbett K (1997). Worksite characteristics and changes in worksite tobacco-control initiatives. Results from the COMMIT study. J. Occup. Environ. Med.

[7] Eisner MD, Smith AK, Blanc PD (1998). Bartenders’ respiratory health after establishment of smoke-free bars and taverns. J. Am. Med. Assn.

[8] Hammond SK (1999). Exposure of U.S. workers to environmental tobacco smoke. Environ. Health Perspect.

[9] Repace J (2004). Respirable particles of carcinogens in the air of Delaware hospitality venues before and after a smoking ban. J. Occup. Environ. Med.

[10] Room R (2004). Smoking and drinking as complementary behaviours. Biomed. Pharmacotherapy.

[11] Bates MN, Fawcett J, Dickson S, Berezowski R, Garrett N (2002). Exposure of hospitality workers to environmental tobacco smoke. Tob. Control.

[12] Jarvis M, Foulds J, Feyerabend C (1992). Exposure to passive smoking among bar staff. Brit. J. Addict.

[13] Maskarinec MP, Jenkins RA, Counts RW, Dindal AB (2000). Determination of exposure to environmental tobacco smoke in restaurant and tavern workers in one US city. J. Expos. Anal. Environ. Epidem.

[14] Siegel M (1993). Involuntary smoking in the restaurant workplace. A review of employee exposure and health effects. J. Am. Med. Assn.

[15] Skeer M, Siegel M (2003). The descriptive epidemiology of local restaurant smoking regulations in Massachusetts: An analysis of the protection of restaurant customers and workers. Tob. Control.

[16] Kiser D, Boschert T (2001). Eliminating smoking in bars, restaurants, and gaming clubs in California: BREATH, the California Smoke-Free Bar Program. J. Public Health Policy.

[17] Macdonald HR, Glantz SA (1997). Political realities of statewide smoking legislation: The passage of California’s Assembly Bill 13. Tob. Control.

[18] Magzamen S, Charlesworth A, Glantz SA (2001). Print media coverage of California’s smokefree bar law. Tob. Control.

[19] Technical Assistance Legal Center Tobacco Laws Affecting California http://talc.phlaw.org/pdf_files/0007.pdf.

[20] Gilpin EA, White MM, White VM, Distefan JM, Trinidad DR, James L, Lee L, Major J, Kealey S, Pierce JP (2003). Tobacco Control Successes in California: A Focus on Young People, Results from the California Tobacco Surveys, 1990–2002.

[21] Weber MD, Bagwell DA, Fielding JE, Glantz SA (2003). Long term compliance with California’s Smoke-Free Workplace Law among bars and restaurants in Los Angeles County. Tob. Control.

[22] Cavan S (1966). Liquor License: An Ethnography of Bar Behavior.

[23] Green PD (2001). Handheld computers as tools for writing and managing field data. Field Method.

[24] Pendragon (2002). Pendragon, 3.2.

[25] Lee JP, Moore RS, Martin SE (2003). Unobtrusive observations of smoking in urban California bars. J. Drug Issues.

[26] Lee JP, Antin TMJ, Moore RS (2008). Social organization in bars: Implications for tobacco control policy. Contemp. Drug Probl.

[27] Moore RS, Lee JP, Martin SE, Johnson TM (2005). Bartender Views on Second-Hand Smoke Related to Tobacco Control Policy.

[28] Hofmann DA, Gavin MB (1998). Centering decisions in hierarchical linear models: Implications for research in organizations. J. Manage.

[29] Snijders TAB, Bosker RJ (1999). Multilevel Analysis: An Introduction to Basic and Advanced Multilevel Modeling.

[30] RTI International (2004). First Annual Independent Evaluation of New York’s Tobacco Control Program: Final Report.

[31] U.S. Census Bureau U.S. Census 2000Online state demographic summary available at http://factfinder.census.gov/servlet/SAFFFacts?+event=Search&+state=04000US06&_lang=en&_sse=on (accessed 2000).

[32] Corrao MA, Guindon GE, Sharma N, Shokoohi DF (2000). Tobacco Control Country Profiles.

[33] O’Hanlon R (1998). The New Irish Americans.

[34] Ryan H, Wortley PM, Easton A, Pederson L, Greenwood G (2001). Smoking among lesbians, gays, and bisexuals: A review of the literature. Am. J. Prev. Med.

[35] Tang H, Greenwood GL, Cowling DW, Lloyd JC, Roeseler AG, Bal DG (2004). Cigarette smoking among lesbians, gays, and bisexuals: How serious a problem? (United States). Cancer Cause. Control.

[36] Moore RS, Antin TMJ, Martin SE, Cochrane M (2006). Take it outside: The changing role of smoking in gay bars.

[37] Moore RS, Lee JP, Antin TMJ, Martin SE (2006). Tobacco free workplace policies and low socioeconomic status female bartenders in San Francisco. J. Epidemiol. Community Health.

[38] Falomir JM, Mugny G, Perez JA, Terry DJ, Hogg MA (2000). Social influence and identity conflict. Attitudes, Behavior, and Social Context: The Role of Norms and Group Membership.

[39] Miller PM, Frederiksen LW, Hosford RL (1979). Social interaction and smoking topography in heavy and light smokers. Addict. Behav.

[40] Pearson M, Michell L (2000). Smoke rings: Social network analysis of friendship groups, smoking and drug-taking. Drugs: Education, Prevention Policy.

[41] Ennett ST, Bauman KE (1993). Peer group structure and adolescent cigarette smoking: A social network analysis. J. Health Soc. Behav.

[42] Hughes J (2003). Learning to Smoke.

[43] Nichter M, Nichter M, Lloyd-Richardson EE, Flaherty BP, Carkoglu A, Taylor N (2006). Gendered dimensions of smoking among college students. J. Adolescent Res.

[44] DiFranza JR, Guerrera MP (1990). Alcoholism and smoking. J. Stud. Alcohol.

[45] Fertig JB, Allen JP (1995). Alcohol and Tobacco: From Basic Science to Clinical Practice NIAAA Research Monograph 30.

[46] Rose JE, Brauer LH, Behm FM, Cramblett M, Calkins K, Lawhn D (2004). Psychopharmacological interactions between nicotine and ethanol. Nicotine Tob. Res.

[47] Klein R (1993). Cigarettes are Sublime.

[48] Satterlund TD, Lee JP, Moore RS, Antin MJ Challenges to implementing and enforcing California’s smoke-free workplace act in bars. Drugs Educ Prev Policy.

